# Prevalence of hypopituitarism and quality of life in survivors of post‐traumatic brain injury

**DOI:** 10.1002/edm2.146

**Published:** 2020-06-05

**Authors:** Meriem Bensalah, Malcolm Donaldson, Malek Labassen, Lyes Cherfi, Mustapha Nebbal, El Mehdi Haffaf, Benaissa Abdennebi, Kamel Guenane, Zahra Kemali, Samia Ould Kablia

**Affiliations:** ^1^ Endocrinology Unit Central Hospital of Army Algiers Algeria; ^2^ Glasgow University School of Medicine Glasgow UK; ^3^ Critical Care Unit Central Hospital of Army Algiers Algeria; ^4^ Neurosurgery Unit Central Hospital of Army Algiers Algeria; ^5^ Nuclear Medicine Unit Central Hospital of Army Algiers Algeria; ^6^ Neurosurgery Unit Salim Zemirli Hospital Algiers Algeria; ^7^ Critical care Unit Salim Zemirli Hospital Algiers Algeria

**Keywords:** growth hormone deficiency, post‐traumatic hypopituitarism, QoL‐AGHDA, quality of life

## Abstract

**Background:**

Hypopituitarism is a recognized sequela of traumatic brain injury (TBI) and may worsen the quality of life (QoL) in survivors.

**Aims:**

To assess the prevalence of post‐traumatic hypopituitarism (PTHP) and growth hormone deficiency (GHD), and determine their correlation with QoL.

**Methods:**

Survivors of moderate to severe TBI were recruited from two Algerian centres. At 3 and 12 months, pituitary function was evaluated using insulin tolerance test (ITT), QoL by growth hormone deficiency in adults’ questionnaire (QoL‐AGHDA), and 36‐item short‐form (SF‐36) health survey.

**Results:**

Of 133 (M: 128; F: 5) patients aged 18‐65 years, PTHP and GHD were present at 3 and 12 months in 59 (44.4%) and 23 (17.29%), 41/116 (35.3%) and 18 (15.5%). Thirteen patients with GHD at 3 months tested normally at 12 months, while 9 had become GHD at 12 months. At 3 and 12 months, peak cortisol was < 500 nmol/L) in 39 (29.3%) and 29 (25%) patients, but <300 nmol/L in only five and seven. Prevalence for gonadotrophin deficiency was 6.8/8.6%, hypo‐ and hyperprolactinaemia 6.8/3.8% and 5.2/8.6%, and thyrotrophin deficiency 1.5/0.9%. Mean scores for QoL‐AGHDA were higher in patients with PTHP at 3 and 12 months: 7.07 vs 3.62 (*P* = .001) and in patients with GHD at 12 months: 8.72 vs 4.09 (*P* = .015). Mean SF‐36 scores were significantly lower for PTHP at 3 months.

**Conclusion:**

Prevalence of PTHP and GHD changes with time. AGHDA measures QoL in GHD more specifically than SF‐36. Full pituitary evaluation and QoL‐AGHDA 12 months after TBI are recommended.

## Graphical abstract

Hypopituitarism is a recognized sequela of traumatic brain injury (TBI) and may worsen the prognosis and quality of life (QoL) of survivors. PTHP and GHD are common after TBI both are linked to QoL one year after injury. AGHDA is a more specific measure of QoL in GHD than SF‐36. Full pituitary evaluation and QoL‐AGHDA 12 months after TBI are recommended.

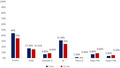

## 1 BACKGROUND

Traumatic brain injury (TBI) represents a serious public health problem and is a leading cause of disability.^1^ Post‐traumatic hypopituitarism (PTHP) was first reported by Cyrian in 1918.^2^ In 2000, two studies reported widely different prevalence for post‐traumatic pituitary deficiency. In 2000, Benvenga et al reviewed 357 cases in a literature review of all the cases reported until 1998.^3^ Since then, more than 20 prospective and retrospective studies have been published and reported an overall prevalence for post‐traumatic pituitary insufficiency of 30%.^4^, ^5^, ^6^, ^7^, ^8^, ^9^, ^10^, ^11^, ^12^, ^13^, ^14^, ^15^, ^16^, ^17^, ^18^, ^19^, ^20^, ^21^, ^22^, ^23^, ^24^, ^25^, ^26^, ^27^, ^28^, ^29^, ^30^ Growth hormone (GH) deficiency is the most frequently reported endocrine consequence of TBI^31^ and results in the adult GH deficiency syndrome. This well‐recognized and distinctive clinical condition comprises lack of energy and fatigue, social isolation, disturbed emotional reactions and social behaviour, poor general health, lack of self‐control, anxiety, decreased vitality, and impaired mood and sense of well‐being as well as problems with sexual relationships.^32^, ^33^ However, these symptoms are also frequently reported by patients who have sustained TBI. The question therefore arises—are such symptoms in TBI survivors related to GH deficiency/PTHP or to the damage caused by the head injury itself? The aim of this study therefore was measure the prevalence of PTHP in TBI survivors and to evaluate their quality of life (QoL) in relation to pituitary status using two scales the Quality of life Assessment for Growth Hormone Deficiency in Adults (QoL‐AGHDA) questionnaire and the Medical Outcomes Survey (MOS) 36‐item short‐form (SF‐36) scale.

## 2 PATIENTS AND METHODS

This prospective study was conducted to evaluate the endocrine and QoL outcome in victims of moderate to severe TBI who had been admitted to the two hospitals in the east of Algiers—Hôpital Central de l’Armée (Central Army Hospital) and Hôpital Salim Zemirli. Between 2010 and 2013 inclusive, 195 patients were admitted to the intensive care or neurosurgical units of the Central Army hospital, and 1249 to the Salim Zemrili hospital, a total of 1443. Eligible patients were recruited by the principal investigator (MB). Inclusion criteria were patients aged 18‐65 years with moderate to severe TBI, defined as Glasgow Coma Scale (GCS) 8‐13 (moderate) and <8 (severe). Exclusion criteria were the presence of pre‐existing endocrine disease, drug or alcohol abuse, chronic illness and previous high‐dose glucocorticoid treatment (eg dexamethasone). The absence of such prescribed glucocorticoids during the acute phase of TBI in our centres and referring hospitals was verified by consulting the hospital records and treatment sheets of the patients. Of note, high‐dose steroids were not normally given to survivors of TBI in the acute phase in our units, given previous work showing that this may worsen outcome.^34^

Acute cortisol evaluation has been presented in a previous publication.^35^ Patients with cortisol < 83 nmol/L in the acute phase or at 3 and 12 months post‐TBI were treated with replacement hydrocortisone, and those with cortisol between 83 and 200 nmol/L were treated if symptomatic and those with cortisol between 200 and 550 nmol/L were counselled and prescribed treatment in the event of major stress. In patients receiving hydrocortisone replacement, treatment was stopped 24 hours before ITT at 3 and 12 months, to avoid any interference with the cortisol assay.

Brain imaging status was classified according to Marshall stratification^36^ as follows: diffuse injury I (no visible pathology); diffuse injury II (midline shift of 0‐5 mm but basal cisterns remain visible, no high or mixed density lesions > 25 cm^3^; diffuse injury III (midline shift of 0‐5 mm, basal cisterns compressed or effaced, but no lesions > 25 cm^3^); diffuse injury IV (midline shift > 5 mm but no lesions > 25 cm^3^); evacuated mass lesion V (any lesion evacuated surgically) and non‐evacuated mass lesion VI (high or mixed density lesions > 25 cm^3^, not surgically evacuated). Marshall stage VI was compared at 3 and 12 months in patients with and without anterior pituitary hormone deficiency.

### 2.1 Hormonal measurements and assays

In the absence of contraindications, for example history of seizures, the insulin tolerance test (ITT) was performed according to consensus guidelines^37^, ^38^ 3 and 12 months after TBI. Insulin was given in the dosage of 0.1 units/kg, taking a glucose nadir of <2.2 mmol/L as representing adequate hypoglycaemia. Sampling was carried out at baseline, 15, 30, 45, 60, 90 and 120 minutes for growth hormone and cortisol, while luteinizing hormone (LH), follicle‐stimulating hormone (FSH), thyroid‐stimulating hormone (TSH), free thyroxine (FT4), testosterone, estradiol, insulin growth factor one (IGF1), adrenocorticotrophic hormone (ACTH) and prolactin were measured at baseline (Table 1).

**TABLE 1:** Pituitary evaluation of survivors of moderate to severe traumatic brain injury at 3 and 12 mo after the initial event. Peak growth hormone and cortisol levels were recorded during the insulin tolerance test, thyroid stimulating hormone, thyroid hormones, gonadotrophins, testosterone and estradiol, and prolactin were recorded at baseline. Post‐traumatic hypopituitarism was defined as the presence of at least one pituitary hormone deficiency

	Evaluation at 3 mo n = 133	Evaluation at 12 mo n = 116
Growth Hormone Deficiency (GHD)	23 (17.29%)	18 (15.51%)
GHD at both 3 and 12 mo	10	10
Normal at 3 mo/GHD at 12 mo	9	
GHD at 3 mo/normal at 12 mo	13	
Treated with GH	0	1
Adrenal insufficiency	39 (29. 5%)	29 (25%)
AI at both 3 and 12 mo	18	18
Normal at 3 mo/AI at 12 mo	11	
AI at 3 mo/normal at 12 mo	21	
Treated with cortisol	7	6
Gonadotrophin deficiency	9 (6.8%)	10 (8.6%)
Males (n = 9)		
Females (n = 0)		
Treated with sex steroids	0	6
Thyrotrophin deficiency	2 (1.5%)	1 (0.9%)
Treated with levo‐thyroxine	2	1
Hypoprolactinaemia	9 (6.8%)	10 (8.6%)
Hyperprolactinaemia	5 (3.8%)	6 (5.2%)
Diabetes insipidus	0 (%)	0 (0%)
Post‐traumatic hypopituitarism	59 (44.4%)	41 (35.3%)
Isolated GHD	15	3
GHD + other pituitary deficiency	8	15
No GHD, other pituitary deficiency	36	23

Abbreviations: AI, adrenal insufficiency; GHD, growth hormone deficiency.

Growth hormone, IGF‐1, cortisol, estradiol, testosterone, prolactin, thyroxine, TSH and prolactin were assayed using Cis‐bio RIA. LH and FSH were assayed using RIA‐MP Biomedicals. Growth hormone deficiency (GHD+) during ITT was defined as a peak value of <3 µg/L (9 mIU/L). IGF‐1 levels were recorded as low if below the age‐related reference range and frankly subnormal if <84 ng/mL (10.97 nmol//L) at any age.

Inter‐assay intervals for cortisol were 5.7‐8 and 1‐6.7, intra‐assay intervals were 5.3‐3.6 and 6‐3.7 for an inferior limit of detection of 3.0 nmol/L. An adequate stimulus for cortisol secretion was taken as ITT‐induced hypoglcaemia of <2.2 mmol/L. The chosen cut‐off for defining adrenal insufficiency (AI) was a cortisol level of <500 nmol/L, in keeping with international recommendation and guidelines.^39^, ^40^ In males, gonadotrophin deficiency was defined as serum testosterone < 8 nmol/L, consistent with our laboratory reference range of 8.2‐34.6 nmol/L and with <8 nmol/L cut‐off recommended by guidelines.^41^, ^42^ In females, gonadotrophin deficiency was defined as estradiol < 60 pmol/L in non‐menopausal women with normal/low gonadotrophins. Measurement of sex hormone‐binding globulin (SHBG) was not available in our laboratory. Hypoprolactinaemia was defined as prolactin < 3.65 µg/L and hyperprolactinaemia as prolactin > 23.7 µg/L in men and >25 µg/L in women, and thyrotrophin deficiency as free thyroxine < 8.25 pmol/L with normal or low TSH. PTHP was considered if the patient had at least one pituitary axis deficiency.

### 2.2 Quality of life assessment

Assessment with QoL‐AGHDA and SF‐36 was performed 3 and 12 months after TBI.

The QoL‐AGHDA consists of 25 items with yes/no answers, acknowledging or denying GHD‐related problems. The maximal score corresponds to 25,^43^ and a poor quality of life is usually attributed to a score higher than 10/25. A French version of the QoL‐AGHDA was validated in 2003 by Leplège et al.^44^ In the present study, QoL scores between patients with and without PTHP and GHD were compared, and the number of patients with PTHP and GHD whose scores exceeded 10/25 were recorded.

SF‐36 is a health‐related quality of life global scale which measures eight subscales: physical function (PF), limitation due to physical health (RP), body pain (BP), general health (GH), vitality (VT), social function (SF), limitation due to emotional health (RE) and mental health (MH). Raw scores are transformed into a scale from 0 to 100 (worst to best). The subscales are grouped into the Physical Component Summary (PCS) which consists of the first four SF‐36 subscales (PF, RP, BP and GH) and the Mental Component Summary (MCS) which consists of the latter four SF‐36 subscales (VT, SF, RE and MH).^45^ Mean scores reported by Leplège et al for the general population for PCS were as follows: 84.45 ± 21, 19 for PF; 81.21 ± 22, 20 for RP; 73.39 ± 23.73 for BP and 69.13 ± 18.57 for GH, and for MCS were as follows: 59.96 ± 18.05 for VT, 81.55 ± 21.41 for SF, 82.13 ± 32.15 for RE and 68.47 ± 17.62 for MH.^45^ Differences between patients with and without PTHP and GHD were compared, and the number of patients with PTHP and GHD at each time point scoring < 1 SD less for each component were recorded.

All the patients were able to answer both questionnaires despite any neurological sequelae from their TBI.

### 2.3 Statistical analysis

This was performed using Student's *t* test of CHI tests for between group comparisons. Comparison of means was with ANOVA or Krusal‐Wallis test, and Pearson coefficient was used for analysing quantitative variables. Cross‐tabulation with chi‐squared test was applied to determine differences in Marshall stage stratification between patients with and without PTHP. Data were analysed using SPSS version 21.

### 2.4 Ethical aspects

The Scientific and Ethics Committees of the central hospital of Army and the Medical Faculty of Algiers approved this study. Consent for recruitment during the acute phase of the study, including sampling for serum cortisol, was obtained from all 277 families on behalf of the patients. Consent for dynamic testing and questionnaire in the survivors at 3 and 12 months was obtained from the patients and their families.

## 3 RESULTS

Between November 2009 and December 2013, 277 patients with moderate or severe TBI who were seen during the acute phase in the two hospitals were enrolled into the study. Of these, 98 patients died, leaving 179 survivors. Sixteen of the survivors were unable to respond to the questionnaire, and 17 could respond to the questionnaire but either could not be tested by ITT or refused dynamic testing, while 13 were lost to follow‐up.

Mean ± SD (range) age at time of TBI in the remaining 133 patients (128 M:5 F) was 32.21 ± 10 (18‐65) years with moderate TBI (GCS 8‐13) in 100 (75.2%) and severe TBI (GCS < 8) in 33 (24.8%). RTA was the most frequent cause, occurring in 48% of patients. Thirty‐five (26.3%) of the patients had multiple injuries and 66 (42%) had intracranial surgery performed. Twenty‐six of the 133 patients studied at 3 months and 22 of the 116 studied at 12 months had isolated intraparenchymal haematoma while hydrocephalus had been present in 13 and 7, respectively.

The 133 patients had received the following drugs during the acute phase of TBI: Midazolam (Hypovnel) (n = 44), Sodium Thiopental (Penthotal) (n = 5), Fentanyl (Fentanyl) or Suphentanyl (Suphenta) (n = 46) and Phenobarbital (Gardenal) (n = 4). No patients in the original cohort of 277 had received Etomidate while none of the five patients who had received Di‐isopropilphenol (Propofol) were included in the present study since two had died, two were unable to complete the questionnaire and one was lost to follow‐up.

Pituitary function was evaluated in all 133 patients at 3 months and in 116 patients at 12 months post‐TBI.

### 3.1 Data relating to pituitary function 3 and 12 months after injury (see Table 1 and Figure 1)

#### 3.1.1 Pituitary hormone deficiency

**FIGURE 1:**
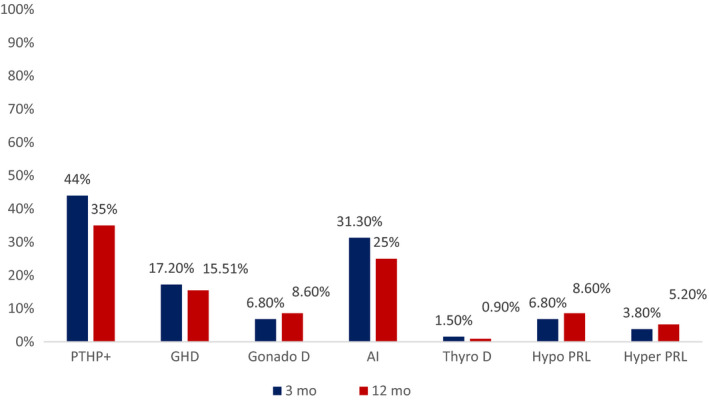
Prevalence of pituitary deficiency at 3 and 12 mo after traumatic brain injury in the 133 and 116 patients, respectively. Data are expressed as percentages. AI, adrenal insufficiency; GHD, growth hormone deficiency; Gn D, gonadotrophin deficiency; Hyper PRL, hyperprolactinaemia; Hypo PRL, hypoprolactinaemia; PTHP+, presence of post‐traumatic hypopituitarism; Thyro D, thyrotrophin deficiency

Table 1 and Figure 1 show the prevalence of pituitary hormone deficiency 3 and 12 months post‐TBI. At 3 months, PTHP was present in 59/133 (44.4%) patients, due to isolated GHD in 15, GHD with one or more other pituitary deficiencies in eight and with normal GH but at least one other pituitary deficiency in 36.

At 12 months, 41/116 (35.3%) patients had PTHP with isolated GHD in three, GHD with one or more other pituitary deficiency in 15 and normal GH but at least one other pituitary deficiency in 23.

### 3.2 Growth hormone deficiency, IGF‐1 levels and body mass index

At 3 months, 23 patients satisfied the criteria for GHD, GH peak < 3 µg/L.

IGF‐1 was below the reference range for age for 4 patients, being frankly subnormal (<84 ng/ml) in 3 patients: 82.6ng/mL (−3.5 SD); 36.9 ng/mL (−6.5 SD) and 57.2 ng/mL (−4.8 SD).

There was a negative but non‐significant correlation between peak GH level on ITT (r = −.09, *P* = .27) and waist circumference (r = −.02, *P* = 0,98) at 3 months. However, peak GH levels were significantly lower in severely obese patients (BMI ≥ 35 kg/m^2^) compared subjects with BMI ≤ 35 kg/m^2^: 4.42 µg/L (n = 13) vs 11.4 µg/L (n = 120) (*P* =< .001). Serum IGF‐1 did not correlate with BMI (r = .04, *P* = .65) but correlated negatively with age (r = −.19; *P* = .02).

At 12 months, 18 patients were categorized as having GHD, peak GH < 3 μg/L.

IGF‐1 was below the reference range for age for four patients, being frankly subnormal (<84 ng/mL) in 2:82.54 ng/mL (−3.9 SD) and 83 ng/mL (−2.9 SD).

Ten patients had GHD both at 3 and 12 months. However, 13 of the patients with peak GH < 3 µg/L at 3 months tested normally at 12 months, while nine who had tested normally at 3 months had become GHD at 12 months. Of the 18 patients with GHD at 12 months, AI was found in nine, gonadotrophin deficiency in two and at least one other pituitary deficiency in 15.

Only one patient received GH replacement therapy after 12 months.

### 3.3 Adrenal insufficiency (see Figure 2A,B)

**FIGURE 2:**
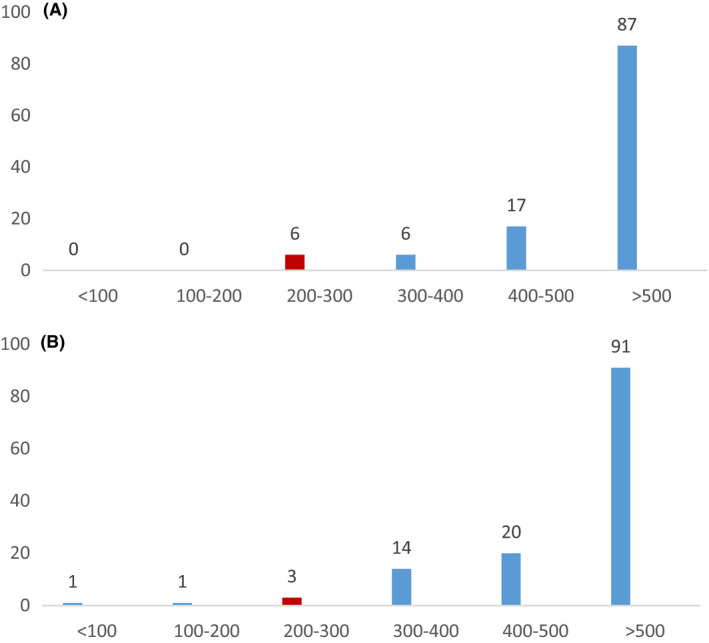
(a) Mean peak cortisol levels during insulin tolerance test at 3 mo in 133 survivors of traumatic brain injury, grouped according to cortisol range (nmol/L). (b) Mean peak cortisol levels during insulin tolerance test at 12 mo in 116 survivors of traumatic brain injury, grouped according to cortisol range (nmol/L)

At 3 months post‐TBI, mean (SD) [range] basal and peak cortisol during ITT (n = 133) was 320.74 (393.29) [4.98‐653.5] and 567.7 (173.1) [17.6‐1384] nmol/L, respectively, with mean basal ACTH 26.5 (0.5‐112) pg/mL. Thirty nine (29.3%) patients had AI, with peak cortisol 400‐<500 nmol/L in 20, 300‐<400 nmol/L in 14, 200‐<300 nmol/L in 3 and <200 nmol/L in 2 (Figure 2A).

At 12 months post‐TBI, basal and peak cortisol values during ITT (n = 116) were 298 (105.2) [77‐543] and 598 (170.2) [222‐1427] nmol/L, respectively, with mean basal ACTH 26.5 (28.3) pg/mL. Twenty‐nine (25%) patients had AI, peak cortisol 400‐<500 nmol/L in 17, 300‐<400 nmol/L in 6, 200‐<300 nmol/L in 6 and <200 nmol/L in none (Figure 2B).

Eighteen patients had AI at both 3 and 12 months, but 21 patients with AI at 3 months tested normal at 12 months, while 11 patients testing normally at 3 months showed AI at 12 months. Of the 25 patients with AI at 12 months, GHD was present in 12 and gonadotrophin deficiency in four.

Nine patients were treated with hydrocortisone during the acute phase of TBI onwards because of basal cortisol < 83 nmol/L (3) or 83‐200 nmol/L (6). At the time of ITT at 3 months, seven patients were still receiving hydrocortisone and continued this medication while two had stopped treatment at their own volition. A further four patients failed the ITT at 3 months and started hydrocortisone replacement. At 12 months, seven patients were still on replacement while four had stopped their treatment. Four of the seven patients failed the ITT and continued replacement treatment while two patients not previously on hydrocortisone failed the test and started treatment. Thus, after 12 months, six patients were receiving hydrocortisone replacement.

### 3.4 Other pituitary hormone deficiencies

Gonadotrophin deficiency was present in 10 (8.6%) male patients and no females at 12 months and TSH deficiency in only one patient. Prolactin levels were subnormal in 10 and elevated in six patients at 12 months. No TBI survivor had diabetes insipidus.

### 3.5 Factors predicting post‐traumatic hypopituitarism at 3 and 12 months (see Figure 3A,B)

Predictive factors of PTHP in bivariate analysis at 3 months were skull base fracture, duration of intubation and initial traumatic imaging according to Marshall stratification, with a significant correlation between the presence and absence of PTHP according to Marshall stages.

**FIGURE 3:**
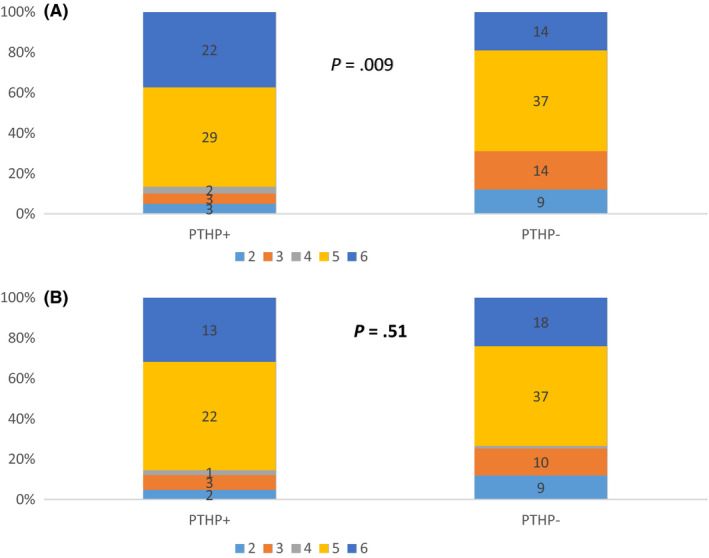
(a) Marshall stratification of post‐traumatic hypopituitary patients (PTHP+) and nonhypopituitary patients (PTHP‐) 3 months after traumatic brain injury. (b) Marshall stratification of post‐traumatic hypopituitary patients (PTHP+) and nonhypopituitary patients (PTHP‐) 12 months after traumatic brain injury

At 12 months, duration of intubation and coma, and multiple injuries predicted PTHP. However, the correlation with Marshal stage stratification was no longer present (Figure 3B).

Severity of brain injury as evaluated by GCS, anaemia, hypotension, papillary status and acute phase surgery did not predict the occurrence of PTHP at either 3 or 12 months after injury.

### 3.6 Quality of life scores 3 and 12 months after injury (Table 2)

Table 2 shows that QoL‐AGHDA scores at 3 months were significantly higher in patients with PTHP than those without (*P* = .0012) but not between GHD and non‐GHD patients (*P* = .65). At 12 months, mean QoL‐AGHDA scores were significantly higher with both PTHP (*P* = .01) and GHD (*P* = .015). QoL scores were >10/25 in 24 and 12 of the 59 patients with PTHP and GHD at 3 months, and in nine and five of the 41 patients with PTHP and GHD at 12 months.

**TABLE 2:** Mean Quality of life Assessment for Growth Hormone Deficiency in Adults (QoL‐AGHDA) scores in patients with and without post‐traumatic hypopituitarism (PTHP) and growth hormone deficiency (GHD) tested at 3 and 12 mo post‐injury

QoL‐AGHDA score	PTHP+	PTHP‐	*P*	GHD+	GHD‐	*P*
3‐month scores (n = 133)	9.75 ± 7.60 (n = 59)	6.04 ± 7.06 (n = 74)	**.0012**	8.18 ± 6.53 (n = 23)	7.48 ± 7.6 (n = 110)	.65
12‐month scores (n = 116)	7.07 ± 6.24 (n = 41)	3.62 ± 4.56 (n = 75)	**.001**	8.72 ± 7.10 (n = 18)	4.09 ± 4.8 (n = 98)	**.015**

Note

*P* < . 05 indicate a significant value. Data are shown as mean ± standard deviation.

The significant values are in bold.

Abbreviations: GHD−, no growth hormone deficiency; GHD+, growth hormone deficiency; PTHP−, no post‐traumatic hypopituitarism; PTHP+, post‐traumatic hypopituitarism.

### 3.7 SF‐36 scores 3 and 12 months after traumatic brain injury (Figure 3A,B)

Figure 3A shows that 3 months post‐TBI, SF‐36 scores were lower than for the general population, particularly concerning physical and general health (RP and GH), social function (SF) and emotional health (RE). Patients with PTHP had lower scores than those without, the difference reaching significance for physical function (PF) (*P* = .014), social function (SF) (*P* = .03), emotional health (RE) (*P* = .02) and mental component summary (MCS) (*P* = .05).

By contrast, there was no difference in the mean scores of the different dimensions of the SF‐36 scale between GHD and non‐GHD patients.

Twelve months after TBI, scores for physical health and social function were still below the mean for the general population at 12 months. However, there was now no significant difference in the mean SF‐36 scores for the different dimensions between patients with and without PTHP and GHD except vitality (VT) scores which were lower in GHD patients (*P* = .02).

## 4 DISCUSSION

TBI is an important public health problem and remains the leading cause of handicap and disability in young adults.^1^ Victims are usually young adult males, with RTA and falls being the most frequent causes.^2^ The prevalence of TBI in Algeria is unknown, but its frequency of RTA is ranked among the top five countries in the world, with 3000 deaths and 25.700 injured during 2016.^46^

The outcome in severe TBI is generally poor, and the presence of neuroendocrine complications may further worsen the prognosis.^1^ PTHP is now well‐recognized as a complication of TBI but, as shown in Table 3, the reported prevalence varies widely between 1% and 83%.^5^, ^6^, ^7^, ^8^, ^9^, ^10^, ^11^, ^12^, ^13^, ^14^, ^15^, ^16^, ^17^, ^18^, ^19^, ^20^, ^21^, ^22^, ^23^, ^24^, ^25^, ^26^, ^27^, ^28^, ^29^, ^30^

**TABLE 3:** Prevalence of post‐traumatic hypopituitarism (PTHP) reported in the literature between 2000 and 2018

Authors/years	N	Prevalence PTHP %	Multiple deficiencies %>3	GHD%	CTD%	GTD%	TTD%
Present study (2020)	133‐116	44‐35	2‐4	17.3‐15.5	29‐25	6.8‐8.6	1.5‐0.9
Kelly (2000)^3^	22	36	9	18	4.5	22.7	4.5
Lieberman (2001)^5^	70	69	17	14.6	45.7	7.1	11.5
Agha (2004)^6^	102	29	5.9	17.6	22.5	11.8	0.98
Bondanelli (2004)^7^	50	54	12	14	0	14	10
Popovic (2004)^8^	67	34	10	14.9	7.5	9	4.5
Aimaretti (2005)^12^	70	22.5	4.2	20	7.1	11.4	5.7
Leal‐Cerro (2005)^14^	170	24	9	5.8	6.4	17	5.8
Schneider (2006)^13^	78	36	NS	10	9	21	3
Tanriverdi (2006)^10^	52	51	9.7	37.7	19.2	7.7	5.8
Herrman (2006)^15^	76	23.7	NS	7.9	2.6	17.1	2.6
Klose (2007)^9^	46	11	7	11	6.5	2.1	2.1
Bushnik (2007)^18^	64	93	44	39	60	14	19
Bavisetty (2008)^19^	70	43	5	16	0	10.5	0
Washter (2009)^17^	53	24.5	1.8	1.8	3.7	15	5.6
Srinivasan (2009)^20^	18	58.8	23.5	17.6	50	0	20.6
Kleindienst (2009)^26^	23	83	30	39	0	48	0
Van Der Eaden (2010)^21^	107	14	NS	0.93	5.6	6.5	0.93
Kokshoorn (2011)^22^	112	5.4	0.8	2.6	1.7	0.8	0
Wilkinson (2012)^23^	26	42	NS	19.2	0	11.5	0
Prodam (2013)^24^	54	27.8	7.4	22.2	NS	NS	NS
Nemes (2014)^25^	126	57.1	NS	39. 7	10.3	23	16.7
Klose (2015)^44^	426	20	2	6. 6	4	5.4	0.5
Silva (2015)^27^	166	31	NS	21	10	12	8
Alavi (2015)^28^	47	21.3	NS	9.1	2	8	0
Kumar (2016)^29^	32	25	NS	12.5	3.12	12.5	6.25
Giuliano (2017)^30^	23	NS	NS	34.7	NS	NS	4.3

Abbreviations: CTD, corticotrophin deficiency; GHD, growth hormone deficiency; GTD, gonadotropin deficiency; NS, not stated; TTD, thyrotrophic deficiency.

In the current study, a PTHP prevalence of 44.4% was found at 3 months, falling to 34.3% at 12 months. Our 3‐month data are in keeping with the studies of Bavisetty,^19^ Aimaretti^12^ and Schneider.^13^ Reasons for the relatively high frequency of PTHP in our study compared with that of Klose et al (11%) could be partly explained by smaller patient numbers (46) and milder TBI in Klose's study in which 22 (50%) of patients had minimal CT changes.^9^

Of note, we excluded patients with mild TBI (GCS > 13) because we considered that this population is at low risk of developing PTHP.

The 12‐month prevalence of PTPH in this study is close to that reported by Schneider,^13^ Popovic^8^ and Agha.^6^ The wide range of prevalence of 11%‐56% reported in other studies^9^, ^10^, ^13^, ^19^ could reflect a combination of confounding variables such as: inclusion/exclusion criteria, timing of evaluation, TBI severity, type of stimulation tests used for evaluating corticotrophin and somatrophin functions and the selected hormone cut‐offs. Such confounders limit the analytical power of meta‐analysis and reviews.^47^ For this reason, it is not possible at present to give a ‘true’ prevalence figure for PTHP. For this to be achieved, international consensus would be required in order to agree the thresholds used to define anterior pituitary insufficiency, the type of tests and assay methods used, CT findings, GCS threshold, and the interval(s) between TBI and assessment.

As with other studies, we found that individual patients with PTHP at 3 months can show resolution by 12 months, attributable to resolution of the initial vascular and ischaemic insults to the pituitary.^48^ In keeping with this, we found a significant correlation between Marshall stratification and PTHP at 3 months, whereas this was no longer present at 12 months, consistent with pituitary recovery in some patients. Additionally, we show that some patients with normal pituitary function at 3 months may develop PTHP, attributable to progressive damage through mechanisms such as autoimmune post‐traumatic hypophysitis.^49^ The differences in GHD and AI prevalence at 3 and 12 months could also reflect the confounding influence of variable intra‐patient reproducibility of the ITT, even though this is the gold standard for assessment of the somatotroph and corticotroph axes.

The prevalence of AI in our study 3 and 12 months post‐TBI is relatively high at 31.6% and 25% but these figures conceal much lower values for moderate as opposed to mild AI, with only 5/133 (4%) and 6/116 (5%) patients showing peak cortisol values of <300 nmol/L.

Administration of drugs during the acute phase of TBI is an unlikely cause of AI 3 and 12 months later, given the half‐life of the agents used. Moreover, of the 133 patients tested at 3 months none had received Propofol (an inhibitor of steroidogenesis) or Etomidate, the only medication in which inhibition of cortisol synthesis has been clearly established.

Predictive factors of PTHP after TBI in this study were as follows: duration of intubation and coma, acute brain imaging (eg Marshall stage) and skull base fracture at 3 months, and duration of coma and intubation, and polytraumatic injury at 12 months. Failure to show the severity of TBI as a predictive factor, in contrast to other studies,^9^, ^13^, ^19^ could be related to the high mortality of patients with low GCS, in whom dynamic endocrine testing was not possible, and also to the exclusion of mild TBI. Moreover, initial GCS may not necessarily predict PTHP since moderate TBI if associated with intracranial bleeding and swelling can induce hypothalamic or pituitary dysfunction, while even relatively mild TBI (GCS 11‐13) may result in hypopituitarism because of the anatomical position and fragile vascularisation of the pituitary gland and stalk.

GHD is the most frequently reported anterior pituitary deficiency when mild AI (peak cortisol 300‐500 nmo/L) is excluded. This finding is explained by the position of the somatrophs in the lateral wings of the anterior lobe and their exclusive vascularization by the superior pituitary artery. The frequency of GH deficiency in our study 3 months post‐TBI was near to 18%, similar to the 16% and 22.8% prevalence reported by Bavisetty^19^ and Aimaretti.^12^ Twelve months after TBI, the frequency of GHD was 15.5%, which is similar to other studies.^3^, ^6^, ^12^, ^19^ The wide variation of 2.6% and 39.7% in GHD prevalence reported in other studies could reflect relatively few centres using the ITT, the gold standard in evaluating growth hormone secretion, different cut‐offs (eg 3 µg/L) for growth hormone secretion, and patient age and BMI.^3^, ^6^, ^9^, ^22^, ^25^ The negative correlation between IGF1 values and age found in this study can be explained by the physiological decline of growth hormone secretion with age^32^ whereas the lack of influence of BMI on GH secretion in response to ITT, except in severe obesity, probably reflects the strong effect of the latter on stimulating GH production.

The link between QoL and adult GHD patients without TBI is well established. Sanmati et al studied 926 patients with adult GHD and found a mean QoL‐AGHDA score of 9.4 compared with 5.49 in the general population.^50^ Data from the Pfizer International Database, KIMS, also showed worse QoL in patients with GHD from all causes.^51^

By contrast, the link between on QoL in patients with TBI and GHD is more contentious. Data from KIMS demonstrated that QoL of patients with post‐traumatic GHD assessed by the QoL‐AGHDA scale were worse than that of GHD patients with non‐functioning adenomas and that 794 patients showed significant 5‐point improvement in QoL‐AGHDA scores after one year of GH treatment.^52^ By contrast, a study comparing scores between patients with TBI and nonfunctional adenomas failed to confirm this.^53^

Our study has shown higher mean QoL‐AGHDA scores at 3 months in patients with PTHP compared with those without, although not between those with and without GHD. By contrast, QoL‐AGHDA scores at 12 months were significantly higher in patients with both PTHP and with GHD compared with those without. One explanation for the discrepancy between scores at 3 and 12 months is the influence of other pituitary hormone deficiencies, but patient numbers are insufficient to support this conjecture. Among other studies which have examined QoL‐AGHDA in TBI,^6^, ^19^, ^49^, ^54^, ^55^ that of Klose et al who studied 46 patients (22 mild TBI) is the only one to find a significant difference in QoL scores between patients with and without PTHP and GHD (*P* = .01).^54^

**FIGURE 4:**
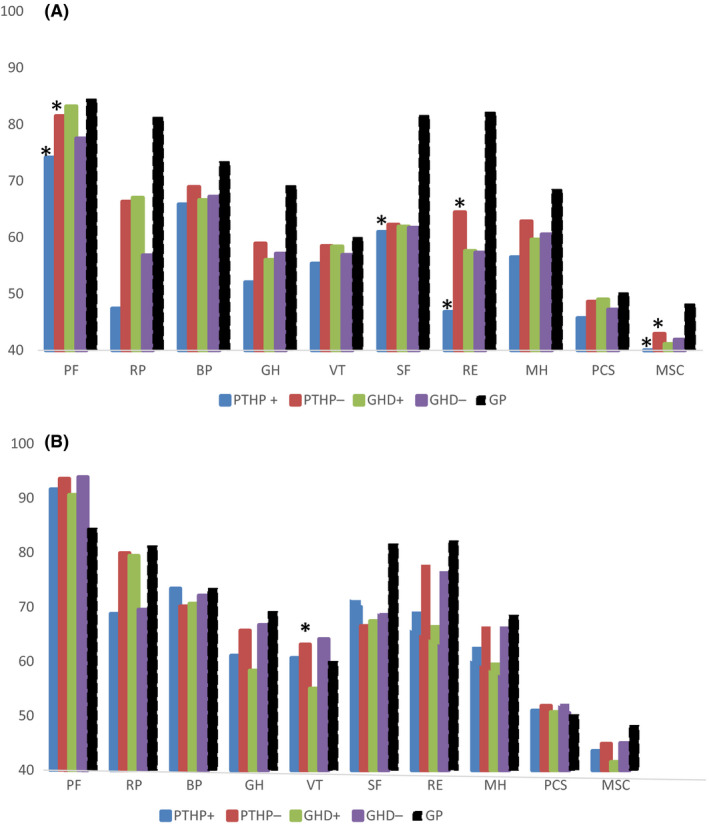
(a, b) Mean scores for the 10 elements of the SF‐36 scale in 133 patients who were assessed 3 mo (a) and 116 patients who were assessed 12 mo (b) after traumatic brain injury. The SF‐36 scale components are shown on the *x*‐axis as: BP, body pain; GH, general health; MH, mental health; MSC, mental component summary—VT, SF, RE and MH combined; PCS, physical component summary—PF, RP, BP and GH combined; PF, physical function; RE, emotional health; RP, physical health; SF, social function; VT, vitality. Scores for each component are shown on the y‐axis. Scores are compared according to pituitary hormone status where PTHP± = post‐traumatic hypopituitarism present/absent; and GHD± = growth hormone deficiency present/absent. * marks significant difference (*P* < .05) between PTHP ± patients; GP, general population

In our study, SF‐36 data show significant differences in physical function, social function and emotional health in patients with and without PTHP at 3 months after TBI, but not for GHD. By 12 months, these differences were no longer evident except for Vitality scores, which were lower in the GHD group. It is noteworthy that scores for Physical Function and Vitality were higher than the general population at 12 months (see Figure 4B), explicable in terms of the marked physical improvement in our younger patients with moderate TBI.

Other studies have used SF‐36 to assess QoL in TBI survivors with and without PTHP, including Kelly, Bavisetty, Nourollahi, Wachter and Kokshoorn who used others scales.^17^, ^19^, ^22^, ^56^ Nouroullahi et al found differences, particularly concerning limitation in physical activity, vitality, social and emotional activities and general health^57^ while Kokshoorn et al found a difference in the general health perception score (*P* = .0016), depression (*P* = .05), social isolation (*P* = .02) and decreased activity (*P* = .027) with post‐traumatic PTHP.^22^ Our findings indicate that SF‐36 assessment 3 months after TBI may be too soon in the recovery period, with some aspects being influenced by pituitary dysfunction which may then resolve, and also by non‐endocrine factors related to the traumatic event, which may also resolve.

Further work is needed in this difficult field, to tease out the effects of GHD and PTHP as opposed to non‐endocrine factors on QoL after TBI, and long term follow‐up is needed to confirm this association. One avenue for future study is to compare QoL in GH‐treated patients with GHD and patients with GHD who remain untreated, and it is our intention to continue monitoring our Algerian cohort of patients.

From a practical point of view, we recommend evaluating pituitary function 3 months after TBI by analysing basal hormones rather than carrying out dynamic testing. At 12 months, full pituitary evaluation should be performed, using the ITT where possible, with QoL‐AGHDA assessment at 12 months, offering GH replacement to patients with GHD and high QoL scores at this stage. SF‐36 assessment at 12 months, although not specific to PTHP, is a valuable tool in the global monitoring of progress in all cases of moderate to severe TBI.

## AUTHOR CONTRIBUTIONS

Doctor Bensalah recruited the patients and, with the help of Dr Labassen, helped with the acute and follow‐up blood samples. The paper was structured and written by Dr Bensalah and Dr Donaldson. Professors Cherfi, Nebbal, Abdennebi and Guenane oversaw the running of the study in their respective critical care and neurosurgery units, enabling access to the patients’ clinical data and laboratory results. Professor El Mehdi was responsible for laboratory analyses while Professors Kemali and Kablia from the two endocrine units oversaw the endocrine testing and analysis and helped with the manuscript. All co‐authors read and approved the final manuscript.

## Data Availability

The data from which this study was written up form part of the MD thesis of the first author, Professor Meriem Bensalah. Access to these data may be requested from the first author, with the proviso that data affecting patient confidentiality will be restricted.
